# Socioeconomic differences in handgrip strength and its association with measures of intrinsic capacity among older adults in six middle-income countries

**DOI:** 10.1038/s41598-021-99047-9

**Published:** 2021-09-30

**Authors:** P. Arokiasamy, Y. Selvamani, A. T. Jotheeswaran, Ritu Sadana

**Affiliations:** 1 International Institute for Population Sciences (IIPS), Govandi Station Road, Mumbai, 400088 India; 2 Department of Maternal, Newborn, Child, Adolescent Health and Ageing, World Health Organization, 20 Avenue Appia, 1211 Geneva, Switzerland; 3 Head, Ageing and Health, Department of Maternal, Newborn, Child, Adolescent Health and Ageing, World Health Organization, 20 Avenue Appia, 1211 Geneva, Switzerland

**Keywords:** Biomarkers, Health care

## Abstract

Handgrip strength, a measure of muscular strength is a powerful predictor of declines in intrinsic capacity, functional abilities, the onset of morbidity and mortality among older adults. This study documents socioeconomic (SES) differences in handgrip strength among older adults aged 50 years and over in six middle-income countries and investigates the association of handgrip strength with measures of intrinsic capacity—a composite of all the physical and mental capacities of an individual. Secondary data analysis of cross-sectional population-based data from six countries from the WHO’s Study on global AGEing and adult health (SAGE) Wave 1 were conducted. Three-level linear hierarchical models examine the association of demographic, socioeconomic status and multimorbidity variables with handgrip strength. Regression-based Relative Index of Inequality (RII) examines socioeconomic inequalities in handgrip strength; and multilevel linear and logistic hierarchical regression models document the association between handgrip strength and five domains of intrinsic capacity: locomotion, psychological, cognitive capacity, vitality and sensory. Wealth quintiles are positively associated with handgrip strength among men across all countries except South Africa while the differences by education were notable for China and India. Work and nutritional status are positively associated with handgrip strength. Our findings provide new evidence of robust association between handgrip strength and other measures of intrinsic capacity and confirms that handgrip strength is a single most important measure of capacity among older persons.

## Introduction

WHO defines healthy ageing as “the process of developing and maintaining the functional ability that enables wellbeing in older age^1^. Functional ability (FA) comprises health-related attributes that enable people to be and to do what they have reason to value, ranging from abilities to meet basic needs, learn and make decisions, get around, build and maintain relationships, and contribute to families, communities and society. Optimizing these abilities reflect the intrinsic capacity of the individual, relevant environmental characteristics, and the interactions between the individual and these characteristics. Intrinsic capacity (IC) is a composite of all the physical and mental capacities—within the mind and body—that an individual can draw on, and at any given point, IC is determined by many factors, including underlying physiological and psychological changes, health-related behaviours, and the presence of disease^1–3^. WHO has operationalized IC as five interrelated domains: vitality, sensory (vision and hearing), locomotor, cognitive capacity and psychological capacity. This paper uses WHO terminologies, intrinsic capacity, and functional ability, and recognizes that previous studies have used different concepts and terms that are not measured or interpreted in the same way.

The measure of handgrip strength is a measure of skeletal muscle function, widely assessed in nationally representative ageing and health studies to assess muscle capacity. The age-associated physiological change in muscle capacity is a well-recognised characteristic of ageing although significant variation at each age is documented^3,4^. WHO identified handgrip strength as a measure of vitality, as noted, one domain of IC, that describes the biophysiological status of an individual and the capacity for maintaining homeostasis in the face of usual daily exposures. Vitality can be conceptualized as the amount of IC that can be retained and be seen as underlying a person’s resilience to challenges, vigour and stamina^3^.

Although loss of grip strength is strongly associated with increasing chronological age, it is also an independent predictor of disability, frailty (significant loss of capacity), morbidity and mortality^5^. Importantly, studies on the association between handgrip strength and health-related outcomes have highlighted handgrip strength as the single most important biomarker of healthy and ageing^5,6^. Muscle weakness was found to be associated with lower cognitive capacity^7–10^, poor psychological outcomes such as geriatric depression, mood and sleep quality and depressive symptoms^11,12^, and as noted, reduced overall intrinsic capacity^13^, including one measure of locomotor capacity, namely gait speed^14^. It also is associated with difficulties in activities of daily living^10^, increased hospitalisation^13^ and overall higher burden of premature morbidity and mortality in older adults^15,16^. These results indicate that handgrip strength, in addition to measuring vitality, could also be a core indicator for monitoring overall intrinsic capacity.

However, the majority of these studies were conducted in high-income countries with very limited studies in low- and middle-income countries (LMICs). A few studies conducted in LMICs showed significant association of handgrip strength with single domains of IC, such as cognition, psychological capacity (includes depression), and other health-related outcomes including mortality. For example, a study conducted in China showed that higher handgrip strength was associated with improved cognitive capacity and a slower decline in cognition with chronologic age^17^. A study by Zhao et al.^18^ based on CHARLS data found a significant association between weaker handgrip strength and increased depressive symptoms among older adults in China. Another study conducted in rural Ghana showed a significant association between weaker handgrip strength and higher mortality among older adults aged 50 and above^19^.

Furthermore, cross-national differences in handgrip strength are strongly evident^20^. Older populations living in European/North American countries have higher handgrip strength than their counterparts in low- and middle-income countries^16,20–23^. Hairi et al.^24^ based on data from the Survey of Health, Ageing and Retirement in Europe (SHARE) document higher handgrip strength is significantly associated with higher levels of education, income, and wealth. Using SHARE data, Cheval et al.^25^ showed significant positive association between socioeconomic status and handgrip strength among older adults in Europe. In addition, a number of studies from higher income countries suggest physical activity is also a significant covariate of handgrip strength^26–28^.

Other studies from low- and middle-income countries such as India, China, South Africa, Sri Lanka, and Indonesia also document a significant association of age, sex, height, and nutritional status with handgrip strength^23,29–31^. However, studies in low-and middle-income countries showed mixed findings on the association between measures of socioeconomic status and handgrip strength. A study among older adults in Indonesia found significant positive association between education and handgrip strength only among men^31^. Whereas, the association between measures of socioeconomic status such as education and wealth quintile and handgrip strength was found significant and positive among older adults in India^29^. In contrast, studies conducted in South Africa and Brazil found no significant association between education and handgrip strength among older adults^26,30^. These findings suggest the need for studies to investigate the association between SES and handgrip strength in each country and understand what is generalizable and what is unique.

Although population ageing is a phenomenon affecting all regions and countries of the world, the pace of change in light of demographic changes (e.g., fertility, mortality and migration) and the response given different policies, epidemiologic transition, resources and investment strategies, is unique to each country. Indisputable is that many low-and middle-income countries are experiencing unprecedented speed of population ageing, with two-thirds of older people worldwide living in middle-income countries. Moreover, China and India, with about a third of the global population, are ageing rapidly. With increasing life expectancy and reduced fertility, the share of people aged 60 and over is projected to increase from 15.2% in China and 8.9% in India in 2015, to 36.5% and 19.4% respectively, by 2050^32^.

However, research on ageing and approaches to develop new, relevant, and efficient measures of healthy ageing of older persons in middle-income country settings such as China and India are limited. To our knowledge, no study has examined the association of individual level socioeconomic status and handgrip strength, or handgrip strength with multiple measures of intrinsic capacity in middle-income countries—countries that have vast within-countries differences, at the individual, household, community, or subnational levels. In low-resource settings, socioeconomic status is an important determinant of overall health and wellbeing mediating by access to health services, nutrition, and social security in older age^29,33^. This study including nationally representative data from six middle-income countries, therefore has two objectives. The first is to explore differences in handgrip strength across countries and by different levels of socioeconomic status in each country, including known demographic and nutritional status correlates. The second is to document the association of handgrip strength with other measures of IC in each of its five domains, to determine whether handgrip strength is a sufficient, single measure of IC, similar to results from high-income countries.

## Results

### Characteristics of the study population

The mean age of men included in the analysis was 61.0 years in South Africa to 64.3 years in Ghana; and for women, 61.1 years in India to 64.3 years in Russia (Supplementary Tables 1 and 2). A higher proportion of participants in China, Ghana and India resided in rural areas. In all countries except Ghana, more than half of the women including in the analysis were not engaged in any form of formal or informal work. The prevalence of underweight was highest in India. Pooled data indicates, although one-third of participants reported at least one chronic disease-, around three-fourths rated their self-reported health status as good (except in Russia), providing evidence that healthy ageing does not require being disease free.

### Cross-national differences in handgrip strength by sex

The mean age-adjusted handgrip strength ranged from 22.8 kg in Mexico to 40.9 kg in South Africa (Table 1). Compared to all other countries, on an average older men and women in India and Mexico had lower handgrip strength (Table 1). Both older men and women in South Africa had higher handgrip strength than older people in other countries. The range of cross-national differences in handgrip strength was substantially greater for men (28.9 kg in Mexico and India and 44.7 kg in South Africa), than for women (18.8 kg in Mexico and 37.6 kg in South Africa). The difference between male–female handgrip strength was higher in Russia and China than other countries. When further disaggregated by 5-year age groups and by sex groups, these differences remain; however, the age-associated decline is stronger for men and women in Russia (Supplementary Figure 1).

**Table 1:** Cross-national differences in mean age-adjusted handgrip strength in kg among older adults aged 50+ stratified by sex, WHO-SAGE Wave 1 (2007/10).

Country	Overall	Men	Women
	Mean (95% CI)	Mean (95% CI)	Mean (95% CI)
China	28.5 (25.5,31.5)	35 (32.1,37.8)	22.9 (20.0,25.8)
Ghana	29.1 (27.7,30.5)	32.7 (31.6,33.9)	24.6 (23.4,25.8)
India	24.38 (23.4,25.3)	28.9 (27.9,29.8)	19.75 (18.4,21.0)
Mexico	22.8 (22.1,23.4)	28.9 (28.1,29.8)	18.8 (18.0,19.5)
Russia	30.9 (29.3,32.5)	41.5 (39.6,43.40)	25.4 (24.2,26.6)
South Africa	40.9 (33.8,48.0)	44.7 (37.3,52.1)	37.6 (30.7,44.6)

### Socioeconomic differences in handgrip strength

The Relative Index of Inequality (RII) scores for years of education and wealth quintile stratified by sex are shown in Table 2. In the age-adjusted model, wealth quintile was significantly and positively associated with handgrip strength among men in all six countries. Educational differences in handgrip strength were significant among men in China and India. Educational attainment and wealth quintile showed significant inequalities in handgrip strength among women in India, China, Russia, and South Africa. In the model fully adjusted for sociodemographic correlates, wealth quintile-based inequalities in handgrip strength were significant among men in all countries except South Africa; whereas educational attainment was significantly associated with higher handgrip strength in India and China. Women in China and Russia with higher educational attainment and upper wealth quintiles had significantly higher handgrip strength. Supplementary Figure 2 shows the predicted handgrip strength estimated from regression by age and wealth quintile for men across six countries. Overall, cross-sectional data documents that wealth differences in handgrip strength narrowed with older age for men in China, Ghana, India, and Russia and for women in Russia and South Africa (Supplementary Figures 2 and 3).

**Table 2:** Relative Index of Inequality in handgrip strength by years of schooling and wealth quintile among older adults aged 50+, WHO-SAGE Wave 1 (2007/10).

Country	Men		Women	
	Schooling	Wealth quintile	Schooling	Wealth quintile
	RII	RII	RII	RII
**Age-adjusted**				
China	3.66*** (2.35,4.98)	4.88*** (3.77,5.99)	0.96* (− 0.044,1.96)	2.4*** (1.51,3.29)
Ghana	− 0.12 (− 1.91,1.66)	3.31*** (1.33,5.28)	1.43 (− 0.55,3.41)	1.27 (− 0.66,3.22)
India	1.61** (0.16,3.06)	4.03*** (2.64,5.43)	1.89* (− 0.093,3.88)	0.97 (− 0.40,2.35)
Mexico	2.37 (− 0.69,5.45)	3.29*** (1.18,5.40)	− 0.96 (− 2.84,0.92)	2.35*** (1.00,3.70)
Russia	2.75 (− 0.54,6.06)	3.82*** (1.37,6.27)	3.04*** (1.27,4.82)	2.45*** (1.11, 3.79)
South Africa	2.21 (− 2.73, 7.16)	4.9** (0.37,9.42)	3.83** (0.10, 7.56)	3.38* (− 0.19,6.96)
**Fully adjusted**^**a**^				
China	3.19*** (1.87,4.51)	4.01*** (2.89,5.14)	0.91* (− 0.097,1.93)	2.13*** (1.23,3.03)
Ghana	0.40 (− 1.35,2.16)	2.32** (0.25,4.39)	1.08 (− 0.86,3.03)	0.17 (− 1.82,2.18)
India	1.07* (− 0.195,2.34)	2.60*** (1.36,3.84)	1.67* (− 0.08,3.43)	0.53 (− 0.70,1.77)
Mexico	1.90 (− 1.29,5.10)	2.70** (0.55,4.86)	− 1.32 (− 3.24,.60)	1.77** (0.37,3.16)
Russia	2.61 (− 0.74,5.98)	2.5* (− 0.07,5.07)	2.6*** (0.84,4.37)	1.38*** (0.004,2.76)
South Africa	1.74 (− 3.21,6.70)	1.92 (− 2.86,6.70)	3.61* (− 0.18,7.41)	1.57 (− 2.10,5.25)

*CI* confidence interval.

^a^Regression analyses were adjusted for age, place of residence, BMI, work status, self-rated health and multimorbidity.

***p < 0.001, **p < 0.005, *p < 0.01.

### Association between socio-demographic measures and handgrip strength

Across the six countries, increasing age was associated with lower handgrip strength among men and women (Supplementary Tables 2 and 3). Older men not engaged in formal or informal work had weaker handgrip strength in India, Mexico, and Russia. Working women had higher handgrip strength in South Africa. Older men who were underweight had lower handgrip strength in China, Ghana, India, Mexico, and South Africa. In China, Ghana and South Africa, older women who were underweight had lower handgrip strength. The association between multimorbidity and handgrip strength was inconsistent. While older men and women with three and more chronic conditions had lower handgrip strength in Russia and South Africa, the association between multimorbidity and handgrip strength was statistically weak in India, China, and Ghana. Poor self-rated health among men was associated with significantly lower handgrip strength in China, India, Russia, and South Africa. Similarly, older women who reported poor self-rated health had lower handgrip strength in India, China, and Russia.

### Association between handgrip strength and measures of intrinsic capacity

Handgrip strength showed significant positive association with measures of cognitive capacity among both men and women in all six countries (Table 3). Handgrip strength was inversely associated with depression in Ghana, India, and Russia. In China, Ghana, Russia and South Africa, higher handgrip strength among men was negatively associated with perceived stress, a second measure of the psychological domain. A similar association was found among women in China, India, Mexico, and Russia. Higher handgrip strength was associated with significantly greater gait speed, a measure of locomotor capacity, in China, Ghana, and Russia. The association between handgrip strength and sensory function measured by visual impairment was significant and negative in China, Ghana, India, Russia, and South Africa, even when many adults used a corrective aid. In China, Ghana, India, Russia, and South Africa, the association between handgrip strength and lung function, an alternative measure of vitality, was significant and positive.

**Table 3:** Association of handgrip strength (kg) with selected measures of intrinsic capacity among men and women aged 50 + in six middle-income countries, WHO-SAGE Wave 1 (2007/10).

	Outcomes	China	Ghana	India	Mexico	Russia	South Africa
**Men**							
Locomotion^a^	Walking speed	− 0.013*** (− 0.013,− 0.010)	− 0.03** (− 0.05,− 0.005)	0.04*** (0.02,0.05)	0.011 (− 0.02,0.042)	− 0.031** (− 0.05,− 0.006)	− .024** (− 0.04,− 0.001)
Psychological^b^	Depression	1.00 (0.98,1.02)	0.93*** (0.92,0.95)	0.97** (0.96,0.99)	1.00 (0.95,1.05)	0.97* (0.94,1.00)	0.99 (0.98,1.01)
Psychological^a^	Perceived stress	− 0.11*** (− 0.16,− 0.05)	− 0.09*** (− 0.17,− 0.02)	− 0.004 (− 0.10,0.09)	− 0.02 (− 0.20,0.15)	− 0.24*** (− 0.35,− 0.13)	− 0.09** (− 0.17,− 0.02)
Cognition^a^	Cognition score	0.09*** (0.06, 0.12)	0.13*** (0.10,0.17)	0.12*** (0.08,0.15)	0.08** (0.006,0.16)	0.17*** (0.12,0.23)	0.08*** (0.05,0.11)
Sensory^b^	Vision loss	0.97*** (0.97, 0.98)	0.97*** (0.96,0.98)	0.99** (0.98,0.99)	0.97** (0.95,0.99)	1.0 (0.99,1.02)	0.99* (0.98,1.00)
Vitality^a^	Lung function (FVC)	0.011*** (0.009, 0.013)	0.019*** (0.015,0.024)	0.016*** (0.004,0.02)	0.004 (− 0.01,0.024)	0.027*** (0.007,0.046)	0.008*** (0.004,0.012)
**Women**							
Locomotion^a^	Walking speed	− 0.014*** (− 0.01,− 0.01)	− 0.05*** (− 0.08,− 0.02)	0.06*** (0.04,0.08)	0.04** (0.006,0.08)	− 0.055*** (− 0.08,− 0.022)	− 0.005 (− 0.02,0.015)
Psychological^b^	Depression	0.99 (0.97,1.01)	0.90*** (0.88,0.92)	0.97*** (0.95,0.99)	0.97* (0.94,1.00)	0.97*** (0.95,0.99)	1.0 (0.99,1.01)
Psychological^a^	Perceived stress	− 0.12*** (− 0.18,− 0.06)	− 0.06 (− 0.14,0.02)	− 0.12** (− 0.22,− 0.018)	− 0.27*** (− 0.48,− 0.07)	− 0.26*** (− 0.37,− 0.15)	0.001 (− 0.06,0.07)
Cognition^a^	Cognition score	0.08*** (0.05,0.11)	0.10*** (0.06,0.13)	0.07*** (0.04,0.11)	0.07* (− 0.007,0.15)	0.19*** (0.13,0.25)	0.13*** (0.10,0.16)
Sensory^b^	Vision loss	0.97*** (0.97,0.98)	0.98** (0.97,0.99)	0.99 (0.98,1.00)	1.00 (0.98,1.02)	0.99 (0.98,1.01)	0.98*** (0.98,0.99)
Vitality^a^	Lung function (FVC)	0.008*** (0.006,0.010)	0.013*** (0.008,0.018)	0.005 (− 0.007,0.019)	− 0.03** (− 0.05,− 0.006)	0.009 (− 0.009,0.029)	0.010*** (0.006,0.013)

*FVC* forced vital capacity.

All regression analyses were adjusted for age, place of residence, years of schooling, wealth quintile, work status, body mass index, self-rated health and multimorbidity.

^a^Results are presented in coefficient with 95% confidence interval.

^b^Results are presented in odds ratio with 95% confidence interval.

***p < 0.001, **p < 0.005, *p < 0.01.

## Discussion

Addressing our first objective, findings of this study confirm substantial cross-national differences in mean handgrip strength for men and women. Among the six countries, older adults aged 50 and over in India and Mexico had much lower handgrip strength compared to their counterparts in the other four countries of South Africa, Russia, Ghana, and China. Such cross-national variations in handgrip strength have been reported previously across the globe suggesting lower handgrip strength in low, middle, and high income countries^16,20^. A study based on older adults from India and the United States found older adults in India had lower handgrip strength^21^. Studies have shown cross-national differences in handgrip strength may be shaped by differences in stature and body size^19^. A few studies have investigated differences by race and ethnicity^34^ however differences most likely reflect within-country heterogeneity in individual socioeconomic factors, nutrition, diet and health behaviour and environmental characteristics reflecting the social determinants of health^33,35^.

That being noted, identifying pathways that lead to lower hand grip strength and policy options to increase equity, is highly relevant. For example, in relation to nutritional status, studies have shown a substantial proportion of older adults in India were underweight (38%), anaemic and experiencing food insecurity (17%)^36,37^. In addition, the prevalence of Vitamin D deficiency was reported to be higher in South Asian countries such as India^38^. This is consistent with other studies that documented a significant relationship between Vitamin D deficiency with measures of intrinsic capacity including lower handgrip strength and gait speed^39^, and outcomes specific to older adults, such as sarcopenia^40^. Moreover, anaemia in older populations particularly women, is common in India and in other LMICs^37^; studies also document its significant association with sarcopenia and handgrip strength^41^.

Unsurprisingly, increasing age showed a consistent inverse association with handgrip strength in each of the six countries. In addition, the variations in handgrip strength at each age are also documented across the six countries (Supplementary Figure 1). Men have on an average, higher handgrip strength than women^3,23,26^. Differences between men and women in handgrip strength was notable across all countries but this difference was smaller in Ghana and South Africa in comparison to other countries. These differences by gender include biological factors related to stature, as well as social and economic determinants that men and women experience differentially. However, reflecting cross-sectional data, the age-associated decline was faster among men consistent with a previous study analysing European populations^42^.

Across the six countries, underweight older adults had lower handgrip strength, similar to a previous study in Indonesia^31^. The findings of our study are important for countries like India and Ghana where a larger proportion of older adults are underweight and have lower handgrip strength. Underweight is shown to be a significant predictor of health outcomes such as anaemia, osteoporosis, reduced cognitive function, depression, and common illnesses in both developing and high income countries^36^. In addition, supporting results of a previous study^31^ older adults who were not engaged in work had lower handgrip strength across the six countries and those who reported poor self-rated health had lower handgrip strength in India, China, and Russia. Longitudinal data are needed to disentangle the determinants and consequences of lower hand grip strength.

Our study reports significant wealth-based inequalities in handgrip strength, particularly among men. The results are consistent with previous studies mainly from high-income countries which document a strong relationship between wealth and handgrip strength compared to other factors such as education and work status^24,29^. The stronger effect of wealth on handgrip strength in old age also confirms wealth as a more relevant measure of socio-economic status than education, particularly in low resource settings^24^. Although the accumulation of wealth takes place across the life course, the benefits of greater wealth are stronger in the later stage of life^42^. For example, higher socioeconomic status has been associated with several advantages such as better access to diet and nutrition^33^. Higher wealth promotes better health outcomes including handgrip strength through intake of diverse nutrition-rich foods. We found a stronger effect of wealth in young old ages of 50–60 among men, but this effect narrowed with older ages over 60, supporting literature that suggests the role of mortality selection and age-as-leveller hypothesis that socioeconomic differences in health weakens with age^43,44^.

Implications of these findings for public policy require further discussion in each context. Nevertheless, there are indications on what can improve the lives of older adults, including policies and interventions promoting improved nutrition that reach older populations in low-resource settings. Existing interventions, such as increasing protein intake, can improve handgrip strength^27^. Further, measures to reduce the inequity of opportunity for appropriate nutrition, for example by targeting individuals with lower socio-economic status, are also necessary to optimize healthy ageing^34^.

Addressing our second objective, our study documents in six middle-income countries, a robust and comprehensive association of handgrip strength with measures of intrinsic capacity. These correspond to each of the five important domains identified by WHO: locomotion, psychological, vitality and cognitive capacity supporting the findings of previous fragmented studies^12–14,17,45,46^. For example, a study conducted among older adults aged 60 and above in Colombia showed significant association between handgrip strength and measures of intrinsic capacity such as vitality, sensory, cognition, and psychological capacities^13^. Our findings of inverse association of handgrip strength with depressive disorders, sensory impairments, and positive association with measures of cognition and gait speed are also consistent^12–14,17,45,46^.

Handgrip strength is a significant marker of intrinsic capacity, its interrelated domains, and is essential for daily functioning. Weaker handgrip strength is a measure of sarcopenia which reflects poor intrinsic capacity and contributes to lower functional ability of older adults^47,48^. In addition, a growing body of literature suggests a significant association between handgrip strength and cardiometabolic disease risk^49^ which further increases the rate of cognitive decline and depending upon the environment, can negatively impact functional ability^50^. Focusing on cognitive capacity, findings from this cross-sectional study show a consistent and positive association between handgrip strength and cognition in six middle income countries. This extends the generalizability of results from studies mainly from high-income countries that reported a significant association between handgrip strength and cognitive capacity. Longitudinal analysis in LMICs, however is needed to confirm whether those with higher handgrip strength experiencing slower cognitive decline^7,9^ and improved psychological health, for example by lowering the risk of depression^13^ and disability^10^. The positive association between handgrip strength and cognitive capacity suggests the role of fluid intelligence (e.g. comprehension, reasoning and problem solving)^51^ and nutritional status^9^ and underlines what is good for the body is also good for the mind^52,53^.

Lastly, the healthy ageing looks at the whole person in their unique environment. Describing and improving functional ability, intrinsic capacity, and environments, the three components of healthy ageing, represents a paradigm shift in thinking about older people and ageing. Information on intrinsic capacity, measured through five domains, provides an important basis to describe comprehensively the capacities of older adults irrespective of disease status. This comprehensive assessment is highly relevant for person centred interventions. Along with enabling environments, these capacities can interactively help to improve the functional ability of the older populations in low- and middle-income country settings. Our results suggest on one hand, handgrip strength is an important measure of overall intrinsic capacity, and on the other, approaches to improve handgrip strength such as through interventions that improve nutritional status that reach all older adults who would benefit, are important for longevity, increasing equity, and promoting healthy ageing^46^.

## Strengths and limitations

The main strength of this study is twofold. It is the first study that documents the pattern of socioeconomic differences in handgrip strength in six middle-income countries and provides cross-national comparative results by age and sex. These results not only provided new insights about the significance of handgrip strength as a marker of overall intrinsic capacity, but also increased our understanding of determinants of handgrip strength, including what is generalizable across low-, middle- and high-income countries and those that are particularly important for populations with lower socioeconomic status.

The main limitation reflects that data is cross-sectional and results do not distinguish between determinants and impacts, which limits causal interpretation. Second, while we investigated the association of handgrip strength with socio-economic status and different measures of each domain of intrinsic capacity, we did not account for different levels of socio-economic status across countries. Recent studies based on longitudinal data from high-income countries have documented possible bi-directional association of the relative effect of handgrip strength on changes in wealth and income, was overall greater than the corresponding effect of income and wealth on health changes^54^. Other studies suggested a positive association between handgrip strength and work participation and economic activity^55^ which contributes to economic wellbeing in old age. Future studies using longitudinal datasets are needed to understand the direction of the association between socioeconomic status and handgrip strength, and how this may differ by gender or other markers of social position. Lastly, similar to other studies based on survey data, findings of our study are subject to possible self-reported bias in reporting of health outcomes. For instance, the prevalence of chronic diseases (arthritis, stroke, hypertension, angina pectoris, diabetes mellitus, asthma, chronic lung disease), and edentulism were based on self-reported data. These limitations notwithstanding, this study sheds important new insights.

## Conclusion

Our study provides new insights of significant wealth-based inequalities in handgrip strength among older adults across six middle-income countries with substantial cross-national differences. In addition, the study provides new evidence of the robust association of handgrip strength with measures of intrinsic capacity across five domains of locomotion, cognitive capacity, psychological, vitality and sensory confirming the multi-dimensional potential of using handgrip strength as a single indicator of intrinsic capacity and a stronger confirmation ageing. Our findings extend the importance of handgrip strength to monitor the progress in healthy ageing in middle-income countries. We conclude by noting that the WHO Guidelines on Integrated Care for Older People (ICOPE) advocate improving physical and mental capacity a comprehensive approach tailored to the specific needs and goals of each older adults—including multimodal exercise, nutritional interventions, and cognitive stimulation, supported by appropriate health and social care systems and service providers^56,57^.

## Methods

### Source

This study uses cross-sectional, population-based survey data from six countries: China, Ghana, India, Mexico, the Russian Federation, and South Africa from WHO’s Study on global AGEing and adult health (SAGE) Wave 1 (conducted during 2007–2010). A multistage cluster sampling strategy was adopted in all countries except Mexico. SAGE included representative samples of persons aged 18–50 years and over in each country with a smaller representative sample of adults aged 18–49 years in each country for comparison. This study included on older adults aged 50 and above in 6 countries (n = 33,878). Household-level and person-level analysis weights were calculated for each country, which include sample selection and a post-stratification adjustment. Detailed information can be accessed from Kowal et al.^58^.

SAGE measures are comparable with other studies from low-, middle- and high-income countries such as the US Health and Retirement Study (HRS) and the family of similar studies such as the Survey of Health, Ageing and Retirement in Europe (SHARE), the English Longitudinal Study of Ageing (ELSA) and the China Health and Retirement Survey (CHARLS). Face to face interviews were conducted to obtain data on sociodemographic characteristics, work history, lifestyles, health risk factors, self-reported and symptomatic assessment of chronic conditions, subjective health, quality of life, cognitive functioning and other domains of IC, disability, and healthcare utilization. In addition, performance or assessed measures of health and anthropometric measures such as height, weight, handgrip strength, lung function, hypertension, waist and hip circumference, timed walk, and vision test, were collected. A detailed description and documentation of data are described elsewhere^58^.

### Measures

#### Handgrip strength

In SAGE, handgrip strength was measured in both the hands using a Smedley Hand Dynamometer (Scandidact Aps, Denmark). The measurement was taken in sitting position with hands dropped to the side. Respondents were asked to keep their upper arm against their body and bend their elbow to 90° with palm facing in (like shaking hands). Subsequently, respondents were asked to squeeze the dynamometer as hard as possible for a few seconds. Overall, two measurements were taken for each hand. In the analysis, we considered the best of the four measurements^59^. Since handgrip strength is the main outcome measure in this study, we excluded missing cases in the analysis (n = 2750), with a final sample of 31,128 for analysis.

### Measures of intrinsic capacity

#### Locomotion

##### Gait speed

In SAGE survey, 4 m gait speed was assessed as a measure of locomotion capacity. Participants were asked to complete the 4-m distance (one attempt) in a normal pace and were permitted to use any mobility aids and the time (in seconds) taken to complete 4 m taken in the analysis^60^. For older adults who used a mobility aid (cane or walker, for example), this was instead a measure of functional ability and is not distinguished in this analysis from IC.

#### Cognition

##### Cognitive ability

In the analysis, we generated a standardised cognitive index with four items-verbal fluency, verbal recall, digit span forward and digit span backward combining these variables covering three domains of cognition using principal components analysis and finally converted index score ranges from 0 to 100, higher scores represent higher cognitive functioning. Detailed description about the construction of cognition index is provided in the supplementary file.

#### Psychological

##### Depression

Depression was assessed through a set of symptomatic questions based on the World Mental Health Survey version of the Composite International Diagnostic Interview^61^. Diagnosis of major depressive episode was derived from an algorithm that accounted for reporting symptoms of depression during the past 12 months. The detailed symptomatic questions and algorithm used are provided in the supplementary file Table S5.

**Perceived stress (control)** was assessed on a five-point scale based on the following question “How often have you felt that you were unable to control the important things in your life?” options were (1) Never, (2) Almost never, (3) Sometimes, (4) Fairly often and (5) Very. **Perceived stress (coping)** was assessed with the following question “How often have you found that you could not cope with all the things that you had to do?” options were (1) Never, (2) Almost never, (3) Sometimes, (4) Fairly often and (5) Very often. In the analysis, we generated a composite perceived stress score index variable based on two questions using factor analysis with polychoric correlations. The scores ranged from 0 to 100, higher scores indicating higher perceived stress^62^.

#### Vitality

##### Lung function

Forced Vital Capacity (FVC) measured in litres were used in the study. Lower scores indicated weaker lung capacity. In SAGE, three measurements were taken, and in the analysis, we considered the best one.

#### Sensory

##### Vision impairment

Visual acuity was measured for both near and distance vision with best possible corrections in each eye using a tumbling “E” logMAR chart. Measured near and distance visual acuity was classified into normal vision (0.32–1.6 decimal) and low vision (0.01–0.25 decimal)^63^. In this study, a respondent was categorised with low vision if they had either low near or distance vision in one or both eyes. For older adults who used a correct aid (spectacles or contact lens, for example), this was instead a measure of functional ability and is not distinguished in this analysis from IC.

### Demographic and socioeconomic factors

The sociodemographic covariates included in the study are **age** (years), **sex** (male and female), **place of residence** (rural and urban), **marital status** (currently married and others), **education** (no schooling-category 1), 1–4 years (category-2), 5–9 years (category-3), and 10 + years (category-4), with the exception of Russia where education is categorised as 0–9 years (category-1), 10–12 years (category-2), 13–15 years (category-3) and 16 + years (category-4). Work status is categorised into currently working and not working/never worked. Wealth quintiles represent household economic status assessed using an index of household assets ranging from possessions, amenities and construction type. Principal Component Analysis was used to generate a wealth index and categorised into five categories (quintiles) ranging from poorest to richest within each country. List of wealth variables included in the index is provided in the Supplementary file Table 7.

### Other health indicators

#### Body mass index (BMI)

BMI was categorized as < 18.5 kg/m^2^ (underweight), 18.5–24.9 kg/m^2^ (normal weight), 25.0–29.9 kg/m^2^ (overweight), 30.0 + kg/m^2^ (obesity)^64^.

### Self-rated health (SRH)

In the SAGE survey, respondents were asked ‘In general, how would you rate your health today? The response categories were: ‘very good’, ‘good’, ‘moderate’, ‘bad’ and ‘very bad’. We combined, ‘bad’ and ‘very bad’ health categories to represent poor self-rated health.

### Chronic diseases (multimorbidity)

Multi-morbidity is defined as the presence of one or more chronic health condition at the time of data collection. In this analysis, we have included eight chronic health conditions: arthritis, stroke, hypertension, angina pectoris, diabetes mellitus, asthma, chronic lung disease and edentulism. Detailed information about the assessment of chronic diseases is described in the supplementary file Table S2.

### Statistical analyses

First, we assessed the association of socioeconomic status, multi-morbidity and handgrip strength using three-level linear hierarchical models, with state/province at the highest level, Primary Sampling Unit (PSU) at the second level, and individuals at the first level. A regression-based age-adjusted handgrip strength was estimated for cross-national comparison. We used the interaction between age and wealth quintile for men and women to understand the age and wealth gradient in handgrip strength. Secondly, the association of handgrip strength with measures of intrinsic capacity in five domains were examined. In view of substantial within country heterogeneity in population characteristics across the six countries, we used multilevel regression models. Three-level linear hierarchical regression models were estimated to assess the linkages of handgrip strength with cognitive capacity, lung function, gait speed, and perceived stress. Further, multilevel logistic regression models examine the association of handgrip strength with depression and visual impairment. We adjusted for ethnicity in all the regression analysis for South Africa. All analyses were conducted in STATA 15.0.

### Relative index of inequality (RII)

RII was used to document the inequalities in handgrip strength by household wealth quintiles. RII is a regression-based inequality measure which accounts for the population distribution across different categories to understand the distribution of the outcome. First, we generated Ridit score for education categories and wealth quintile (poorest, poorer, middle, richer and richest). Subsequently, we used the Ridit score in the multivariable regression models to obtain the RII score.

### Ethics declaration

The SAGE study was approved by the Ethics Review Committee (RPC146), World Health Organization, Geneva, Switzerland, and the Institutional Review Boards of six participating countries.

## Supplementary Information

Supplementary Information.

## Data Availability

The data can be freely downloaded from the WHO’s website through the following link: http://apps.who.int/healthinfo/systems/surveydata/index.php/catalog/sage.
