# Supplementary material for: Socioeconomic differences in handgrip strength and its association with measures of intrinsic capacity among older adults in six middle-income countries
Source: Sci Rep. 2021 Sep 30;11:19494. doi: 10.1038/s41598-021-99047-9 (PMC8484588; doi:10.1038/s41598-021-99047-9)
Supplement: Supplementary file 1 — Supplementary Information. [file 41598_2021_99047_MOESM1_ESM.docx]

**Title Page**

**Socioeconomic Differences in Handgrip Strength and its Association with Measures of Intrinsic Capacity among Older Adults in Six Middle-Income Countries**

P Arokiasamy PhD^1^, Y Selvamani PhD^`1,^ A T Jotheeswaran, PhD^2^, Ritu Sadana ScD^2^

^1^International Institute for Population Sciences (IIPS),

Govandi Station Road, Mumbai. 400088. India

^2^Department of Maternal, Newborn, Child, Adolescent Health and Ageing, World Health Organization, 20 Avenue Appia, Geneva 1211, Switzerland.

^*^Address correspondence to: Y Selvamani, International Institute for Population Sciences (IIPS), Govandi Station Road, Mumbai. 400088.

Email: [selvinsw@gmail.com](mailto:selvinsw@gmail.com) or [selvamani@iips.net](mailto:selvamani@iips.net)

**Supplementary Figure 1Mean handgrip strength by 5-year**

**age group for men and women in six middle countries**

**Supplementary Table 1 Characteristics of the study population for men (weighted %, unweighted n), WHO-SAGE Wave 1 (2007/10)**

| Characteristics | China  (n= 5,733) |  | Ghana |  | India |  | Mexico |  | Russia |  | South Africa |
| --- | --- | --- | --- | --- | --- | --- | --- | --- | --- | --- | --- |
|  |  |  | (n= 2,200) |  | (n= 3,230) |  | (n= 791) |  | (n= 1,103) |  | (n= 1,103) |
|  | n (%) |  | n (%) |  | n (%) |  | n (%) |  | n (%) |  | n (%) |
| **Residence** |  |  |  |  |  |  |  |  |  |  |  |
| Urban | 2,584 (41.9) |  | 829 (40.9) |  | 766 (27.5) |  | 557 (73.4) |  | 758 (70.7) |  | 969 (65.7) |
| Rural | 3,149 (58.2) |  | 1,371 (59.1) |  | 2,464 (72.5) |  | 234 (26.6) |  | 345 (29.3) |  | 501 (34.3) |
| **Marital status** |  |  |  |  |  |  |  |  |  |  |  |
| Married | 5,129 (90.6) |  | 1,830 (84.89) |  | 2,835 (91.4) |  | 619 (90.0) |  | 906 (78.4) |  | 1,109 (80.4) |
| Others | 602 (9.44) |  | 360 (15.1) |  | 395 (8.61) |  | 152 (10.0) |  | 197 (21.7) |  | 342 (19.6) |
| **Body mass index** |  |  |  |  |  |  |  |  |  |  |  |
| Underweight | 243 (3.98) |  | 325 (14.9) |  | 1,132 (39.3) |  | 6 (0.47) |  | 11 (1.27) |  | 71 (4.26) |
| Normal weight | 3,761 (64.5) |  | 1,340 (59.7) |  | 1,700 (50.7) |  | 229 (20.3) |  | 295 (26.9) |  | 445 (28.2) |
| Overweight | 1,472 (27.6) |  | 371 (18.3) |  | 294 (8.26) |  | 341 (57.2) |  | 530 (44.8) |  | 432 (28.6) |
| Obesity | 224 (3.88) |  | 132 (7.21) |  | 59 (1.82) |  | 174 (22.0) |  | 221 (27.0) |  | 487 (38.9) |
| **Work status** |  |  |  |  |  |  |  |  |  |  |  |
| Currently working | 2,735 (54.2) |  | 1,629 (72.8) |  | 1,943 (66.1) |  | 352 (61.6) |  | 458 (50.7) |  | 533 (40.5) |
| Not working/never worked | 2,976 (45.8) |  | 562 (27.2) |  | 1,287 (33.9) |  | 419 (38.4) |  | 645 (49.3) |  | 925 (59.4) |
| **Schooling** |  |  |  |  |  |  |  |  |  |  |  |
| Category 1 | 780 (13.0) |  | 974 (43.8) |  | 1,062 (30.6) |  | 117 (11.3) |  | 252 (18.6) |  | 278 (21.4) |
| Category 2 | 1,457 (26.2) |  | 173 (8.56) |  | 756 (22.7) |  | 324 (36.1) |  | 426 (44.9) |  | 254 (25.1) |
| Category 3 | 2,280 (42.9) |  | 185 (8.39) |  | 598 (19.2) |  | 233 (38.6) |  | 291 (28.3) |  | 365 (29.6) |
| Category 4 | 1,131 (17.8) |  | 823 (39.2) |  | 804 (27.5) |  | 83 (14.0) |  | 111 (8.21) |  | 268 (23.9) |
| **Wealth quintile** |  |  |  |  |  |  |  |  |  |  |  |
| Poorest | 1,120 (16.3) |  | 414 (16.1) |  | 517 (17.3) |  | 142 (12.3) |  | 155 (11.5) |  | 261 (20.1) |
| Poorer | 1,177 (19.0) |  | 404 (17.4) |  | 596 (18.4) |  | 177 (26.3) |  | 172 (14.0) |  | 298 (20.5) |
| Middle | 1,195 (20.9) |  | 428 (19.6) |  | 588 (18.8) |  | 137 (12.1) |  | 205 (16.2) |  | 241 (13.5) |
| Richer | 1,193 (23.8) |  | 451 (21.5) |  | 713 (20.9) |  | 170 (19.9) |  | 265 (24.7) |  | 294 (19.7) |
| Richest | 1,034 (20.0) |  | 501 (25.3) |  | 797 (24.5) |  | 165 (29.3) |  | 304 (33.5) |  | 369 (26.2) |
| **Multimorbidity** |  |  |  |  |  |  |  |  |  |  |  |
| No disease | 1,604 (28.3) |  | 691 (29.1) |  | 1,131 (36.9) |  | 175 (23.2) |  | 269 (24.6) |  | 265 (16.9) |
| 1 disease | 2,481 (45.2) |  | 1,008 (47.7) |  | 1,073 (32.6) |  | 318 (45.6) |  | 310 (29.0) |  | 768 (53.4) |
| 2 diseases | 1,102 (18.5) |  | 390 (18.2) |  | 577 (16.3) |  | 204 (22.5) |  | 255 (21.3) |  | 281 (18.5) |
| 3+ diseases | 546 (7.94) |  | 111 (5.07) |  | 449 (14.1) |  | 94 (8.71) |  | 269 (25.1) |  | 156 (11.1) |
| Poor self-rated health (%) | 1,040 (18.8) |  | 296 (14.6) |  | 594 (19.2) |  | 73 (10.7) |  | 233 (14.9) |  | 218 (15.8) |
| Depression (%) | 96 (1.55) |  | 158 (6.96) |  | 496 (17.3) |  | 52 (4.13) |  | 55 (2.45) |  | 64 (4.75) |
| Visual impairment (%) | 1,927 (34.3) |  | 613 (29.1) |  | 1,342 (43.5) |  | 342 (41.2) |  | 467 (45.1) |  | 546 (37.8) |
| Perceived stress M, SD | 30.8, 20.5 |  | 49.0, 20.4 |  | 43.3, 24.49 |  | 20.5, 19.4 |  | 37.7, 20.3 |  | 44.0, 26.0 |
| Time to walk 4 metre M, SD | 4.18, 1.19 |  | 6.73, 8.46 |  | 5.00, 3.93 |  | 4.66, 3.06 |  | 6.37, 3.91 |  | 7.09, 7.87 |
| Lung function (FVC) M, SD | 2.68, .79 |  | 2.51, 1.14 |  | 1.74, 2.72 |  | 3.99, 2.19 |  | .55, 5.44 |  | 2.55, 1.31 |
| Cognition capacity M, SD | 52.4, 11.5 |  | 46.0, 11.1 |  | 41.5, 9.44 |  | 44.87, 10.2 |  | 54.1, 13.1 |  | 47.2, 12.1 |
| Handgrip strength M, SD | 34.7, 11.4 |  | 31.9, 12.8 |  | 28.5, 10.3 |  | 31.9, 8.5 |  | 42.7, 13.0 |  | 41.6, 20.4 |
| Age M, SD | 62.0, 8.61 |  | 64.3, 10.8 |  | 61.4, 8.5 |  | 61.6, 11.3 |  | 61.1, 9.43 |  | 61.0, 9.17 |

M=mean SD=standard deviation

FVC=Forced Vital Capacity

**Supplementary Table 2 Characteristics of the study population for women (weighted %, unweighted n), WHO-SAGE Wave 1 (2007/10)**

| Characteristics | China  (n= 6,530) |  | Ghana |  | India |  | Mexico |  | Russia |  | South Africa |
| --- | --- | --- | --- | --- | --- | --- | --- | --- | --- | --- | --- |
|  |  |  | (n= 1,969) |  | (n= 3,139) |  | (n= 1,174) |  | (n= 1,873) |  | (n= 1,916) |
|  | n (%) |  | n (%) |  | n (%) |  | n (%) |  | n (%) |  | n (%) |
| **Residence** |  |  |  |  |  |  |  |  |  |  |  |
| Urban | 3,339 (49.9) |  | 883 (41.6) |  | 851 (29.5) |  | 870 (85.1) |  | 1,439 (72.6) |  | 1,290 (64.3) |
| Rural | 3,191 (50.1) |  | 1,371 (58.4) |  | 2,288 (70.5) |  | 304 (14.9) |  | 434 (27.4) |  | 626 (35.7) |
| **Marital status** |  |  |  |  |  |  |  |  |  |  |  |
| Married | 5,090 (80.3) |  | 540 (31.0) |  | 1,923 (62.7) |  | 556 (58.9) |  | 849 (46.8) |  | 677 (36.8) |
| Others | 1,434 (19.7) |  | 1,417 (69.0) |  | 1,216 (37.3) |  | 585 (41.1) |  | 1,019 (53.2) |  | 1,196 (63.17) |
| **Body mass index** |  |  |  |  |  |  |  |  |  |  |  |
| Underweight | 294 (4.10) |  | 280 (14.6) |  | 1,035 (36.9) |  | 11 (0.64) |  | 15 (1.30) |  | 57 (2.40) |
| Normal weight | 3,725 (55.2) |  | 962 (49.5) |  | 1,476 (46.2) |  | 252 (21.8) |  | 338 (18.1) |  | 398 (19.9) |
| Overweight | 1,992 (32.4) |  | 411 (21.4) |  | 423 (13.27) |  | 417 (43.9) |  | 691 (41.7) |  | 507 (25.3) |
| Obesity | 477 (8.30) |  | 274 (14.5) |  | 143 (3.67) |  | 433 (33.7) |  | 763 (38.9) |  | 900 (52.39) |
| **Work status** |  |  |  |  |  |  |  |  |  |  |  |
| Currently working | 2,106 (35.2) |  | 1,278 (66.0) |  | 674 (20.9) |  | 173 (18.2) |  | 564 (35.1) |  | 397 (23.6) |
| Not working/never worked | 4,400 (64.8) |  | 684 (34.0) |  | 2,465 (79.1) |  | 968 (81.8) |  | 1,308 (64.9) |  | 1,503 (76.4) |
| **Schooling** |  |  |  |  |  |  |  |  |  |  |  |
| Category 1 | 2,245 (33.4) |  | 1,302 (65.8) |  | 2,204 (73.2) |  | 260 (22.0) |  | 542 (28.0) |  | 472 (26.3) |
| Category 2 | 1,533 (25.7) |  | 157 (8.30) |  | 490 (15.3) |  | 445 (39.1) |  | 655 (34.3) |  | 352 (19.9) |
| Category 3 | 1,815 (28.5) |  | 157 (8.21) |  | 234 (6.5) |  | 315 (31.5) |  | 461 (29.4) |  | 554 (32.2) |
| Category 4 | 860 (12.5) |  | 338 (17.6) |  | 203 (4.89) |  | 101 (7.3) |  | 180 (8.34) |  | 329 (21.6) |
| **Wealth quintile** |  |  |  |  |  |  |  |  |  |  |  |
| Poorest | 1,350 (16.8) |  | 400 (19.6) |  | 509 (19.1) |  | 288 (16.8) |  | 371 (16.8) |  | 377 (20.7) |
| Poorer | 1,295 (18.3) |  | 422 (21.3) |  | 580 (19.5) |  | 242 (25.7) |  | 383 (19.1) |  | 386 (20.6) |
| Middle | 1,288 (20.6) |  | 397 (21.4) |  | 593 (19.5) |  | 204 (20.1) |  | 390 (21.2) |  | 418 (22.1) |
| Richer | 1,320 (23.2) |  | 404 (20.3) |  | 653 (18.4) |  | 223 (13.4) |  | 361 (20.8) |  | 389 (19.2) |
| Richest | 1,254 (21.0) |  | 343 (17.4) |  | 786 (23.5) |  | 216 (24.0) |  | 365 (22.1) |  | 339 (17.5) |
| **Multimorbidity** |  |  |  |  |  |  |  |  |  |  |  |
| No disease | 1,636 (24.2) |  | 445 (22.8) |  | 974 (31.6) |  | 182 (13.5) |  | 316 (20.8) |  | 277 (14.3) |
| 1 disease | 2,718 (42.6) |  | 909 (46.8) |  | 1,107 (33.9) |  | 450 (49.2) |  | 468 (25.7) |  | 920 (46.9) |
| 2 diseases | 1,387 (22.1) |  | 447 (22.2) |  | 626 (20.8) |  | 321 (15.97) |  | 475 (23.7) |  | 443 (23.3) |
| 3+ diseases | 789 (11.2) |  | 168 (8.2) |  | 432 (13.7) |  | 221 (21.3) |  | 614 (29.8) |  | 276 (15.5) |
| Poor self-rated health (%) | 1,491 (24.0) |  | 386 (19.1) |  | 695 (24.6) |  | 149 (22.4) |  | 532 (25.8) |  | 278 (15.3) |
| Depression (%) | 182 (2.51) |  | 220 (11.2) |  | 598 (21.0) |  | 192 (23.7) |  | 197 (11.1) |  | 97 (5.18) |
| Visual impairment (%) | 2,700 (42.5) |  | 738 (37.5) |  | 1,655 (55.2) |  | 568 (53.7) |  | 794 (44.8) |  | 816 (42.9) |
| Perceived stress M, SD | 34.3, 21.8 |  | 51.9, 21.7 |  | 44.3, 25.9 |  | 26.6, 22.49 |  | 44.3, 18.3 |  | 46.0, 26.7 |
| Time to walk 4 metre M, SD | 4.54, 1.48 |  | 7.53, 8.58 |  | 5.63, 4.48 |  | 5.83, 3.31 |  | 8.26, 7.39 |  | 7.76, 9.38 |
| Lung function (FVC) M, SD | 1.96, .65 |  | 1.87, 1.22 |  | .76, 3.43 |  | 3.76, 3.07 |  | .62, 4.77 |  | 2.27, 1.19 |
| Cognition capacity M, SD | 49.4, 11.8 |  | 41.2, 10.4 |  | 34.5, 9.26 |  | 42.4, 9.4 |  | 52.4, 12.7 |  | 44.2, 11.7 |
| Handgrip strength M, SD | 22.1, 10.1 |  | 24.9, 12.1 |  | 18.9, 9.63 |  | 20.6, 6.04 |  | 25.9, 9.2 |  | 33.7, 18.6 |
| Age M, SD | 62.7, 9.02 |  | 64.1, 10.4 |  | 61.1, 8.96 |  | 62.7, 10.4 |  | 64.3, 10.0 |  | 61.8, 9.61 |

M=mean SD=standard deviation

FVC=Forced Vital Capacity

**Supplementary Table 3 Regression results of handgrip strength (kg) among men in six middle-income countries, WHO-SAGE, 2007-10**

| Characteristics | China | Ghana | India | Mexico | Russia | South Africa |
| --- | --- | --- | --- | --- | --- | --- |
|  | β [95% CI] | β [95% CI] | β [95% CI] | β [95% CI] | β [95% CI] | β [95% CI] |
| **Age (years)** | -0.35***(-0.39 -0.32) | -0.25***(-0.30 -0.20) | -0.25***(-0.29 -0.21) | -0.26***(-0.32 -0.20) | -0.45***(-0.53 -0.36) | -0.34***( -.47 -.21) |
| **Marital status** |  |  |  |  |  |  |
| Currently married | ref | ref | ref | ref | ref | ref |
| Otherwise | -0.81**(-1.58 -0.04) | -0.25(-1.53 1.02) | -0.52 (-1.38 0.33) | -1.32*(-2.75 0.11) | -1.28 (-3.04 0.47) | .39 (-1.96 2.75) |
| **Place of residence** |  |  |  |  |  |  |
| Urban | ref | ref | ref | ref | ref | ref |
| Rural | -1.92 (-4.35 0.51) | 0.43 (-1.57 2.44) | 0.78 (-0.33 1.90) | -0.85 (-2.58 0.88) | 1.48 (-1.60 4.60) | -1.37 (-4.62 1.88) |
| **Schooling** |  |  |  |  |  |  |
| Category 1 | ref | ref | ref | ref | ref | ref |
| Category 2 | 1.20***(0.41 1.98) | -1.25 (-3.06 0.55) | 0.43 (-0.34 1.21) | 0.35 (-1.36 2.06) | 1.02 (-0.81 2.86) | -1.33 (-4.43 1.75) |
| Category 3 | 1.75***(0.95 2.55) | -0.35 (-2.11 1.40) | 0.99**(0.11 1.87) | 1.28 (-0.64 3.21) | 1.05 (-0.93 3.04) | -2.18 (-5.21 .85) |
| Category 4 | 2.29***(1.31 3.26) | 0.22 (-1.02 1.48) | 0.60 (-0.31 1.51) | 0.71 (-1.70 3.15) | 2.29*(-0.26 4.85) | 1.02 (-2.77 4.82) |
| **Wealth quintile** |  |  |  |  |  |  |
| Poorest | ref | ref | ref | ref | ref | ref |
| Poorer | 0.88**(0.13 1.63) | 0.76 (-0.75 2.28) | 0.47 (-0.47 1.42) | -0.28 (-2.05 1.49) | 1.61 (-0.74 3.96) | 1.25 (-2.14 4.65) |
| Middle | 2.01***(1.21 2.82) | 2.16***(0.62 3.69) | 0.57 (-0.40 1.55) | -0.59 (-2.49 1.30) | 0.82 (-1.48 3.13) | 3.42 *(-0.33 7.18) |
| Richer | 2.64***(1.80 3.49) | 1.74**(0.12 3.37) | 1.55***(0.54 2.56) | 0.81 (-1.06 2.69) | 3.05***(0.78 5.32) | .91 (-2.97 4.79) |
| Richest | 3.09***(2.12 4.05) | 1.79**(0.02 3.57) | 2.04***(0.97 3.12) | 1.96**(0.05 3.86) | 2.01*(-0.26 4.30) | -0.04 (-4.33 4.24) |
| **Work status** |  |  |  |  |  |  |
| Currently working | ref | ref | ref | ref | ref | ref |
| Not working/  never worked | -0.36 (-0.98 0.25) | -0.85 (-2.26 0.55) | -1.29***(-1.95 -0.64) | -1.37**(-2.53 -0.21) | -2.54***(-4.18 -0.90) | -.21 (-2.74 2.31) |
| **Body mass index** |  |  |  |  |  |  |
| Underweight | -2.32***(-3.47 -1.16) | -3.51***(-4.83 -2.19) | -2.37***(-3.00 -1.74) | -7.89**(-14.4 -1.35) | 2.51 (-3.61 8.64) | -4.73 **( -9.45 -.014) |
| Normal weight | ref | ref | ref | ref | ref | ref |
| Overweight | 1.71***(1.16 2.26) | 1.42**(0.14 2.69) | 1.71***(0.72 2.70) | 2.14***(0.85 3.43) | 0.94 (-0.57 2.45) | 2.21* (-.39 4.81) |
| Obesity | 1.73***(0.50 2.96) | -0.90 (-2.92 1.10) | 5.24***(3.18 7.30) | 1.97**(0.41 3.54) | -0.36 (-2.26 1.52) | 1.89 (-0.81 4.60) |
| **Multimorbidity** |  |  |  |  |  |  |
| No morbidity | ref | ref | ref | ref | ref | Ref |
| 1 disease | 0.25 (-0.31 0.81) | -0.385 (-1.46 0.69) | -0.56*(-1.23 0.10) | -1.46**(-2.92 -0.01) | -0.58 (-2.35 1.18) | -2.50* (-5.36 .33) |
| 2 diseases | -0.16 (-0.87 0.55) | -1.261*(-2.63 0.11) | -1.35***(-2.1 -0.52) | -2.10***(-3.70 -0.57) | 0.25 (-1.70 2.21) | -6.51***(-9.98 -3.03) |
| 3+diseases | -0.24 (-1.18 0.69) | -0.28 (-2.40 1.91) | -0.38 (-1.31 0.55) | -1.27 (-3.31 0.76) | -0.99 (-3.05 1.07) | -6.28***(-10.5 -2.06) |
| **Self-rated health** |  |  |  |  |  |  |
| Good | ref | ref | ref | ref | ref | ref |
| Poor | -1.29***(-1.93 -0.66) | -2.11***(-3.30 -0.93) | -1.80***(-2.65 -1.08) | -0.59 (-2.40 1.30) | -1.33 (-3.04 0.37) | -3.78**(-6.84 -0.72) |
| Random part |  |  |  |  |  |  |
| Region | 4.17 (2.34, 7.41) | 1.18 (0.34, 4.03) | 0.99 (0.45, 2.16) | 1.29 (0.57, 2.93) | 3.94 (2.04, 7.61) | 10.8 (6.66, 17.5) |
| PSU | 4.79 (3.95, 5.80) | 6.24 (5.52, 7.05) | 3.76 (3.36, 4.19) | 2.33 (1.59, 3.39) | 5.42 ( 4.05, 7.27) | 7.89 (6.45, 9.65) |
| SD(Residual) | 8.60 (8.44, 8.76) | 9.91 (9.60, 10.2) | 7.51 (7.32, 7.71) | 7.07 (6.67, 7.49) | 9.85 (9.41, 10.3) | 15.9 (15.3, 16.7) |
| Observations | 5,574 | 2,107 | 3,157 | 722 | 1,031 | 1,102 |

**CI = confidence interval, *** p<.001, ** p<.005, * p<.01**

**SD=standard deviation**

**PSU= primary sampling unit**

**Supplementary Table 4 Regression results of handgrip strength (kg) among women in six middle-income countries, WHO-SAGE, 2007-10**

| Characteristics | China | Ghana | India | Mexico | Russia | South Africa |
| --- | --- | --- | --- | --- | --- | --- |
|  | β [95% CI] | β [95% CI] | β [95% CI] | β [95% CI] | β [95% CI] | β [95% CI] |
| **Age (years)** | -0.24***(-0.26 -0.21) | -0.18***(-0.23 -0.12) | -0.16***(-0.20 -0.12) | -0.18***(-0.22 -0.14) | -0.22***(-0.27 -0.17) | -0.14***(-0.23 -0.05) |
| **Marital status** |  |  |  |  |  |  |
| Currently married | ref | ref | ref | ref | ref | ref |
| Otherwise | -0.18 (-0.68 0.31) | -1.55***(-2.64 -0.47) | -0.38 (-1.03 0.27) | -0.04 (-0.78 0.69) | -0.71*(-1.47  0.03) | -3.90***(-5.76 -2.37) |
| **Place of residence** |  |  |  |  |  |  |
| Urban | ref | ref | ref | ref | ref | ref |
| Rural | -2.07*(-4.16 0.006) | 0.86(-1.00  2.72) | 1.25**(0.11  2.39) | -0.13(-1.54 1.26) | 1.85**(0.012 3.70) | -2.48*(-5.20 .23) |
| **Schooling** |  |  |  |  |  |  |
| Category 1 | ref | ref | ref | ref | ref | ref |
| Category 2 | 0.39 (-0.13 0.91) | -0.56 (-2.29 1.17) | 0.81*(-0.05 1.67) | -0.97**(-1.93 -0.01) | 0.91*(-0.02  1.86) | 3.18***(.87 5.49) |
| Category 3 | 0.56*(-0.01 1.14) | 0.40 (-1.30 2.14) | 1.17*(-0.01 2.37) | -0.68 (-1.70 0.40) | 1.83***(0.75 2.91) | 0.73 (-1.53 3.00) |
| Category 4 | 0.52 (-0.24 1.29) | 0.82 (-0.61 2.25) | 0.62 (-0.83 2.08) | -1.09 (-2.62 0.43) | 1.10 (-0.27 2.49) | 2.97**(0.89 5.85) |
| **Wealth quintile** |  |  |  |  |  |  |
| Poorest | ref | ref | ref | ref | ref | ref |
| Poorer | -0.11 (-0.71 0.49) | -1.23*(-2.66 0.18) | -0.18 (-1.16 0.80) | 0.56 (-0.50 1.62) | 1.26**(0.18 2.35) | 0.572 (-2.09 3.24) |
| Middle | 0.48 (-0.16 1.13) | -0.42 (-1.88 1.04) | 0.38 (-0.62 1.39) | 0.70 (-0.45 1.85) | 1.47**(0.35 2.58) | 0.36 (-2.39 3.12) |
| Richer | 1.23***(0.54 1.91) | -0.17 (-1.73 1.37) | -0.12 (-1.16 0.91) | 1.14*(-0.01 2.29) | 1.00*(-0.14 2.15) | 1.25 (-1.69 4.20) |
| Richest | 1.44***(0.67 2.21) | -0.37 (-2.14 1.39) | 0.43 (-0.64 1.51) | 1.46**(0.24 2.68) | 1.50**(0.29 2.71) | .73 (-2.66  4.13) |
| **Work status** |  |  |  |  |  |  |
| Currently working | ref | ref | ref | ref | ref | ref |
| Not working/  never worked | -0.09 (-0.62 0.42) | -0.50 (-1.61 0.61) | -0.65*(-1.40 0.08) | 0.20 (-0.78 1.19) | -0.91*(-1.86 0.037) | -3.30***(-5.34 -1.26) |
| **Body mass index** |  |  |  |  |  |  |
| Underweight | -1.37***(-2.28 -0.45) | -2.41***(-3.80 -1.01) | -0.50 (-1.17 0.166) | -2.12 (-5.63 1.38) | -0.05 (-3.83 3.73) | -5.13**(-9.7 -.57) |
| Normal weight | ref | ref | ref | ref | ref | ref |
| Overweight | 1.027***(0.60 1.45) | 0.35 (-0.87 1.57) | 0.94**(0.04 1.83) | 2.20***(1.27 3.14) | 0.79 (-0.17 1.76) | 1.56 (-0.63 3.77) |
| Obesity | 1.38***(0.63 2.12) | 2.16***(0.67 3.66) | 0.83 (-0.58 2.26) | 1.67***(0.73 2.61) | 1.87***(0.88 2.85) | 1.06 (-.98 3.11) |
| **Multimorbidity** |  |  |  |  |  |  |
| No morbidity | ref | ref | ref | ref | ref | ref |
| 1 disease | 0.38 (-0.08 0.86) | -0.35 (-1.53 0.81) | 0.29 (-0.41 1.00) | 0.142 (-0.91 1.19) | -0.64 (-1.74 0.46) | -0.85 (-3.16 1.44) |
| 2 diseases | 0.019 (-0.55 0.59) | -1.07 (-2.46 0.30) | 1.37***(0.52 2.21) | 0.14 (-0.98 1.27) | -1.48**(-2.63 -0.33) | -3.05**(-5.67 -0.43) |
| 3+diseases | -0.03 (-0.74 0.68) | -1.75*(-3.65 0.15) | 0.31 (-0.66 1.28) | -0.64 (-1.88 0.58) | -2.13***(-3.34 -0.92) | -4.77***(-7.76 -1.77) |
| **Self-rated health** |  |  |  |  |  |  |
| Good | ref | ref | ref | ref | ref | ref |
| Poor | -0.86***(-1.34 -0.38) | 0.51 (-0.71 1.74) | -1.03***(-1.79 -0.27) | -0.32 (-1.39 0.73) | -1.43***(-2.30 -0.56) | 0.41 (-1.92 2.76) |
| Random part |  |  |  |  |  |  |
| Region | 4.29 (2.47, 7.43) | 1.06 (0.35 3.14) | 1.43 (0.73, 2.80) | 1.74 (1.11 2.71) | 2.25 (0.95, 5.31) | 10.9 (6.79, 17.7) |
| PSU | 4.12 (3.40, 4.99) | 5.64 (4.94, 6.44) | 3.75 (3.32, 4.23) | 2.18 (1.68, 2.83) | 3.88 (2.90, 5.18) | 7.36 (6.24, 8.67) |
| SD(Residual) | 7.47 (7.34, 7.60) | 9.52 (9.20, 9.85) | 7.69 (7.48, 7.90) | 5.44 (5.19, 5.70) | 7.06 (6.82, 7.31) | 14.7 (14.1, 15.2) |
| Observations | 6,352 | 1,889 | 3,052 | 1,066 | 1,769 | 1,601 |

**CI = confidence interval, *** p<.001, ** p<.005, * p<.01**

**SD=standard deviation**

**PSU= primary sampling unit**

**Supplementary Figure 2 Age pattern of handgrip strength by wealth quintile for men in six middle-income countries, WHO-SAGE, 2007-10**

**Supplementary Figure 3 Age pattern of handgrip strength by wealth quintile for women in six middle-income countries, WHO-SAGE, 2007-10**

**Cognitive capacity**

To understand the composite effect of cognition we made a cognitive index combining four variables: verbal fluency, verbal recall, digit span forward and digit span backward.

The composite index of cognition was derived using Principal Components Analysis (PCA), a mathematical tool which helps in creating a composite index using uncorrelated components, where each component captures the largest possible variation in the original variables. Selected raw scores for cognitive tasks were bundled into three domains (digit span, memory and executive functioning) to yield compound cognitive scores. This was done to condense the number of cognitive variables while refining the robustness of the underlying cognitive construct. We followed two steps to make a cognitive index:

*Step 1*: All four variables were in different scales. So first, we standardized these variables. A standardized variable (sometimes called a z-score or a standard score) is a variable that has been rescaled to have a mean of zero and a standard deviation of one. Each case's value on the standardized variable designates its difference from the mean of the primary variable in some standard deviations (of the original variable).

*Step 2*: PCA is a multivariate statistical technique used for extracting from a set of variables those few orthogonal linear combinations that capture the common information most successfully. Further, this index comprises both values, positive and negative. So we converted this index into a 0–100 scale which facilitates easier interpretation of the data. Higher scores indicate better cognitive abilities.

**Chronic diseases**

Among eight chronic diseases included in the study, for arthritis, angina pectoris, asthma, lung disease, SAGE survey provides two types of measures: First, self-reports of the diagnosis of individual diseases, and second is the symptom-based assessment or direct health examination of abovementioned diseases. The specific question asked in SAGE for self-reports is: “Have you ever been diagnosed with/told that you have disease (name of disease)? The analysis considered an individual as positive for these diseases if he/she was found positive in the symptom-based assessment or direct health examination. For stroke, diabetes mellitus and edentulism, we have relied on the self-reports of diagnosed disease. The prevalence of loss of all-natural teeth was assessed based on a specific question “Have you lost all of your natural teeth?” yes and no. In the analysis, respondents who reported ‘yes’ are considered as edentulous. The prevalence of hypertension was based on measured blood pressure (systolic and diastolic) taken with the respondent in a seated position. An average of the second and third of three total readings was used as the outcome. In accordance with WHO/ISH guidelines for the management of hypertension^58^, the threshold for high systolic blood pressure was 140 mm/hg or above, and for diastolic blood pressure 90 mm/hg or above. An individual was considered to be hypertensive if average systolic or diastolic blood pressure readings exceeded either of these thresholds or they reported current treatment for hypertension. Further, we generated a multi-morbidity variable by combining eight chronic diseases: 0 diseases, 1 disease, 2 diseases and 3+ diseases.

**Supplementary Table 5** **Symptoms and algorithms used to derive prevalence of depression in the SAGE Wave 1, 2007/10.**

| **Depression** | 1 | During the last 12 months, have you had a period lasting several days when you felt sad, empty, or depressed? |
| --- | --- | --- |
|  | 2 | During the last 12 months, have you had a period lasting several days when you lost interest in most things you usually enjoy such as personal relationships, work, or hobbies/recreation? |
|  | 3 | During the last 12 months, have you had a period lasting several days when you have been feeling your energy decreased or that you are tired all the time? |
|  |  | If any of the above three questions are yes then following set of questions were asked |
|  | 4 | Was this period [of sadness/loss of interest/low energy] for more than 2 weeks? |
|  | 5 | Was this period [of sadness/loss of interest/low energy] most of the day, nearly every day? |
|  | 6 | During this period, did you lose your appetite? |
|  | 7 | Did you notice any slowing down in your thinking? |
|  | 8 | Did you notice any problems falling asleep? |
|  | 9 | Did you notice any problems waking up too early? |
|  | 10 | During this period, did you have any difficulties concentrating; for example, listening to others, working, watching TV, listening to the radio? |
|  | 11 | Did you notice any slowing down in your moving around? |
|  | 12 | During this period, did you feel anxious and worried most days? |
|  | 13 | During this period, were you so restless or jittery nearly every day that you paced up and down and couldn’t sit still? |
|  | 14 | During this period, did you feel negative about yourself or like you had lost confidence? |
|  | 15 | Did you frequently feel hopeless - that there was no way to improve things? |
|  | 16 | During this period, did your interest in sex decrease? |
|  | 17 | Did you think of death, or wish you were dead? |
|  | 18 | During this period, did you ever try to end your life? |
|  | Algorithm | To ascertain the depression from this set of questions two set of variables were computed. First set was based on the questions1, 2, 3, 4, 5 and 16. From this set three variables were computed taking values 0 and 1: a) first variable takes value 1 if response to any of questions 1, 4, and 5 was yes. b) second variable takes value 1 if question 2 or 16 has response yes. c) the third variable takes value 1 if question 3 has response yes. The second set of variables was based on questions 6, 7, 8, 9, 10, 11, 12, 13, 14, 15, 17 and 18. From these questions seven variables were computed. a) first variable takes value 1 if response to questions 14 or 15 is yes. b) second variable takes value 1 if response to questions 12 or 13 is yes. c) third variable takes value 1 if questions 17 or 18 has response yes. d) fourth variable takes value 1 if questions 7 or 10 has response yes. e) fifth variable takes value 1 if response to questions 11 is yes. f) sixth variable takes value 1 if response to questions 8 or 9 is yes. g) seventh variable takes value 1 if the response to question6 is yes. These newly created variable from the respective sets were added to obtain two new variables: first consisting sum of first set of variables (maximum value 3) and second consisting sum of second set of variables (maximum value 7). Based on these two variables, a respondent is said to suffer from depression if he has value for the first variable to be 2-plus and the value for second variable to be 4-plus. |

**Supplementary Table 6 Symptoms and algorithms used to derive prevalence of individual chronic diseases in the SAGE Wave 1, 2007/10**

| **Disease name** | **Questions No** | **Question Text and algorithm to ascertain diseases** |
| --- | --- | --- |
| **Arthritis** | 1 | During the last 12 months, have you experienced, pain, aching, stiffness or swelling in or around the joints (like arms, hands, legs or feet) which were not related to an injury and lasted for more than a month? |
|  | 2 | During the last 12 months, have you experienced stiffness in the joint in the morning after getting up from bed, or after a long rest of the joint without movement? |
|  |  | If yes to question 2 |
|  | 3 | How long did this stiffness last? (1) less than 30 mins or 2) more than 30 mins |
|  | 4 | Did this stiffness go away after exercise or movement in the joint? 1.yes 2. No |
|  | **Algorithm** | **If response for questions 1 & 2 was yes and for 3 and 4 was first option then the respondent was said to have arthritis** |
| **Angina** | 1 | During the last 12 months, have you experienced any pain or discomfort in your chest when you walk uphill or hurry? |
|  | 2 | During the last 12 months, have you experienced any pain or discomfort in your chest when you walk at an ordinary pace on level ground? |
|  |  | If yes to question 2 |
|  | 3 | What do you do if you get the pain or discomfort when you are walking?(1 Stop or slow down 2 Carry on after taking a pain relieving medicine that dissolves in your mouth 3 Carry on walking) |
|  | 4 | If you stand still, what happens to the pain or discomfort? (1 Relieved 2 Not relieved) |
|  | 5 | Apart from these questions respondents were asked to identify the points of pain in the upper part of the body (excluding head) with help of a picture depicting upper parts of the body |
|  | **Algorithm** | **If the response to questions 1 & 2 was yes and for 3 & 4 it was first option & from the question 5 pain was in the left upper part of body the person was said to have angina** |
| **Lung diseases** | 1 | During the last 12 months, have you experienced any shortness of breath at rest? (while awake) |
|  | 2 | During the last 12 months, have you experienced any coughing or wheezing for ten minutes or more at a time? |
|  | 3 | During the last 12 months, have you experienced any coughing up sputum or phlegm for most days of the month for at least 3 months? |
|  | **Algorithm** | **a respondent was ascertained to have chronic lung disease if his response was yes to question 1 or yes to questions 2 and 3 both** |
| **Asthma** | 1 | During the last 12 months, have you experienced Attacks of wheezing or whistling breathing? |
|  | 2 | During the last 12 months, attack of wheezing that came on after you stopped exercising or some other physical activity? |
|  | 3 | During the last 12 months, a feeling of tightness in your chest? |
|  | 4 | During the last 12 months, have you woken up with a feeling of tightness in your chest in the morning or any other time? |
|  | 5 | During the last 12 months, have you had an attack of shortness of breath that came on without obvious cause when you were not exercising or doing some physical activity? |
|  | **Algorithm** | **A respondent was said to suffer from asthma if he responded yes question1 and yes to any of the subsequent questions (2-5).** |

**Supplementary Table 7** List **of items used for calculation of household wealth, WHO-SAGE, 2007-10**

| **Question No.** | **Item details** |
| --- | --- |
| q0700 | Can you please tell me how many rooms there are in your home? |
| q0701 | How many chairs are there in your home? |
| q0702 | How many tables are there in your home? |
| q0703 | How many cars are there in your household? |
| q0704 | Does your home have electricity? |
| q0705 | Does anyone in your household have a bicycle? |
| q0706 | Does anyone in your household have a clock? |
| q0707 | Does anyone in your household have a bucket? |
| q0708 | Does anyone in your household have a washing machine for clothes? |
| q0709 | Does your household or anyone in your household have a dishwasher? |
| q0710 | Does anyone in your household have a refrigerator? |
| q0711 | Does anyone in your household have a fixed line telephone? |
| q0712 | Does anyone in your household have a mobile/cellular telephone? |
| q0713 | Does anyone in your household have a television? |
| q0714 | Does anyone in your household have a computer? |
| q0715 | Does anyone in your household have moped/scooter/motorcycle? |
| q0716 | Does anyone in your household have live-stock (cattle only)? |
| q0717 | Does anyone in your household have sewing machine? |
| q0718 | Does anyone in your household have radio/transistor/tape recorder? |
| q0719 | Does anyone in your household have bullock cart? |
